# Undiagnosed Genetic Muscle Disease in the North of England: an in Depth Phenotype Analysis

**DOI:** 10.1371/currents.md.37f840ca67f5e722945ecf755f40487e

**Published:** 2013-05-21

**Authors:** Elizabeth Harris, Steve Laval, Judith Hudson, Rita Barresi, Liesbeth De Waele, Volker Straub, Hanns Lochmüller, Kate Bushby, Anna Sarkozy

**Affiliations:** Institute of Genetic Medicine, Newcastle University, Newcastle upon Tyne, UK; Institute of Genetic Medicine, Newcastle University, Newcastle upon Tyne, UK; Institute of Genetic Medicine, Newcastle University, Newcastle upon Tyne, UK; NSCT Diagnostic & Advisory Service for Rare Neuromuscular Diseases, Muscle Immunoanalysis Unit, Dental Hospital, Newcastle upon Tyne, UK; Institute of Genetic Medicine, Newcastle University, Newcastle upon Tyne, UK; Department of Paediatrics, University Hospitals KU Leuven, Belgium; Institute of Genetic Medicine, Newcastle University, Newcastle upon Tyne, UK; Institute of Genetic Medicine, Newcastle University, Newcastle upon Tyne, UK; Institute of Genetic Medicine, Newcastle University, Newcastle upon Tyne, UK; Institute of Genetic Medicine, Newcastle University, Newcastle upon Tyne, UK

## Abstract

Advances in the molecular characterisation of genetic muscle disease has been rapid, as demonstrated by a recent analysis of these conditions in the north of England by Norwood et al (2009), in which a genetic diagnosis was achieved for 75.7% of patients. However, there remain many patients with suspected genetic muscle disease in who a diagnosis is not obtained, often despite considerable diagnostic effort, and these patients are now being considered for the application of new technologies such as next generation sequencing. This study aimed to provide an in-depth phenotype analysis of undiagnosed patients referred to the Northern region muscle clinic with suspected genetic muscle disease, with the intention of gaining insight into these conditions, identifing cases with a shared phenotype who may be amenable to collective diagnostic testing or research, and evaluating the strengths and limitations of our current diagnostic strategy. We used two approaches: a review of clinical findings in patients with undiagnosed muscle disease, and a hierarchical cluster analysis to provide an unbiased interpretation of the phenotype data. These joint approaches identified a correlation of phenotypic features according to the age of disease onset and also delineated several interesting groups of patients, as well as highlighting areas of frequent diagnostic difficulty that could benefit from the use of new high-throughput diagnostic techniques. Correspondence to: anna.sarkozy@ncl.ac.uk

## Introduction

Genetic muscle diseases are a genetically and clinically heterogeneous group of disorders characterised by progressive weakness and wasting of skeletal muscles^1^. The molecular pathogenesis of many of these diseases has been elucidated, and more than 200 genes have now been implicated in inherited neuromuscular disorders^2^. The northern region muscle clinic (NMC) provides a clinical service to approximately 1200 adults and children with a range of genetic muscle diseases in the north of England (see figure 1 in Norwood *et al*, 2009^3^). A recent analysis of the population attending the NMC found that a molecularly confirmed genetic diagnosis was achieved in 75.7% of our patients, representing thirty-one different genetic muscle diseases^3^ . This illustrates the significant advances that have been made in the characterisation of these diseases yet also reveals that for many patients with suspected genetic muscle disease a definitive genetic diagnosis remains elusive. For individuals without a molecular diagnosis prognosis is uncertain and genetic counselling only speculative. This lead us to perform an analysis of patients with suspected but currently undiagnosed genetic muscle disease from the Northern region with the intention of gaining better insight into these phenotypes and highlighting areas of diagnostic difficulty. This analysis is pertinent given the increasing availability of next generation sequencing (NGS) which may provide a genetic diagnosis in these patients, but will require a systematic approach to ensure the benefits of this technology is cascaded to all undiagnosed patients.

## Methods

**Clinical data collection and review**

The northern region population in this study is defined by the same primary care trust-determined boundaries as described by Norwood *et al *(2009)^3^ . The NMC is a tertiary referral centre for children and adults considered to have a genetic muscle disease, and is based at the Institute of Genetic Medicine in Newcastle-upon-Tyne. Patients with acquired causes of muscle disease, channelopathies, neuromuscular junction and peripheral nerve disorders are referred and seen within other dedicated clinical services in the region. Patients included in the study were identified from the database of attendance at the NMC.

Patients attending NMC underwent standardised clinical diagnostic protocols including creatin kinase (CK) measurement, standardised physiotherapy assessment with strength testing, and cardiac and respiratory assessment in addition to more invasive evaluations such as muscle biopsy and electromyography (EMG), depending on an individual’s age, comorbidities, severity of illness, and preference^4^ . Molecular investigation included direct sequencing and/or linkage analysis of known neuromuscular disease genes/loci. Genes most commonly excluded at the time of data collection (2010) are listed in table 1. Practical limitations including financial cost mean that individual genes are selected for screening on a case-by-case basis for each referral, tailored to the clinical and histopathological phenotype of the patient. Evaluations are performed on a 6-12 monthly basis for all patients, and include general clinical evaluation, physiotherapy assessment and lung capacity assessment with forced vital capacity (FVC) and peak expiratory flow (PEF1) monitoring. Clinical information pertaining to all patients in the NMC was collated into a clinical database and analysed by a single investigator (LH) (table 2). Patients with a confirmed molecular diagnosis at time of completion of this study were excluded from the study. The frequency of clinical features was reviewed according to the age of disease manifestation.

**Hierarchical cluster analysis**

A hierarchical cluster analysis was performed using Gene Cluster v3.0. Clinical features, selected based on their frequency and potential diagnostic relevance, were categorised and converted into numerical identifiers with a corresponding specific colour. For example, the category of inheritance pattern was converted as follows: no other affected family members = 1 = black, consistent with recessive inheritance = 2 = dark red, consistent with dominant inheritance = 3 = medium red. For the purposes of this analysis some categories were combined to reduce the number of categories per clinical feature. This was based on our experience that performing this analysis on data where several clinical features have more than three categories resulted in a high degree of complexity in the results, making interpretation difficult. In addition, although skin pathology and any other relevant clinical features were recorded in the database they were not included in the cluster analysis to reduce the complexity of the resulting analysis.

The data was loaded into Gene Cluster 3.0 (by Michiel de Hoon http://bonsai.hgc.jp/~mdehoon/software/cluster/software.htm) and subjected to hierarchical clustering using an unweighted average linkage of the Pearson correlation of Euclidean distances separating clusters. Dendrograms were drawn using TreeView v1.1.5r2 (by Alok Saldanha; http://jtreeview.sourceforge.net).

**Ethics statement**

This work represents a retrospective case series report for patients seen and samples analyzed under diagnostic agreements and no formal ethical committee approval was requested.

**Table 1: Genes frequently analysed in undiagnosed patients attending Northern Region muscle clinic:** 

Disease group	Genetic analysis
Dystrophinopathies	*DMD* gene **
Fascioscapulohumeral muscular dystrophy	deletion in subtelomeric region of 4q35
LGMD 1A-G	*MYOT, LMNA, *or *CAV3 *gene **
LGMD2A-O	*CAPN3, DYSF, SGCG, SGCA, SCCB, SGCD, TCAO, FKRP, POMT1/2, FCMD*, *POMGNT1 *or* ANO5* gene **
Emery Dreifuss muscular dystrophy (X-linked, autosomal dominant or recessive)	*EMD, LMNA or FHL1 *gene** **
Congenital myopathies	*SEPN1, RYR1, ACTA1, TPM2, TPM3, NEB *gene**
Congenital muscular dystrophies	*LAMA2, FKRP, POMT1, POMT2,POMGNT1, LARGE, Fukutin *gene **
Myofibrillar myopathies	*DES, MYOT, CRYAB, ZASP, FLNC, BAG3 *gene**
Myotonic dystrophies	*DMPK* or *ZNF9* gene**
Spinal muscular atrophies	*SMN1 *gene**
Collagenopathies	*COL6A1, COL6A2, *or *COL6A3 *gene**
FHL1 associated myopathies	*FHL1* gene**
Inclusion body myopathy (with/withoutPagets disease of bone and frontotemporal dementia)	*GNE, VCP* gene**

**Table 2: Clinical features present in undiagnosed patients as recorded in database for phenotype analysis:** 

**Clinical Feature**	**Categories for phenotype analysis**
Inheritance	Sporadic (no affected relatives) Consistent with recessive inheritance Consistent with dominant inheritance
Age of disease onset	Congenital onset (birth to 2 years) Childhood (2 – 16 years) Adult onset (>16 years)
Sex	Male Female
Pattern of weakness	Proximal only Proximal>distal Proximal and distal Distal>proximal Distal only No weakness
	Upper only Upper >lower Upper and lower Lower>upper Lower only No weakness
Serum CK (IU/L)	Normal 250-999 1,000-2,999 3,000 - 9,999 >10,000
Joint Contractures	Absent Present at 2 or less joint sites Present at 3 or more joint sites
Rigid spine	Present Absent
Scoliosis	Present Absent
Respiratory dysfunction	Present (defined as forced viral capacity (FVC) of <70% predicted for age and height, fall in FVC of >10% sitting / lying, or use of ventilatory support) Absent
Cardiac dysfunction	Present (dysrhythmia, structural abnormality or cardiomyopathy) Absent
Skin abnormalities	Any skin pathology recorded
Learning difficulties	Present Absent
Opthalmoplegia	Present Absent **
Other features	Any other relevant features also recorded

## Results

**Composition of the cohort of undiagnosed muscle patients**

A total of 119 patients (70 male, 49 female) from 112 families were selected from the database and included the study. Patients had been followed up for a mean of 8 years. The mean age of patients was 36 years, and five patients had died during follow up. The largest affected family comprised three affected siblings. Muscle biopsy had been performed in 105 subjects (88.2 %). Muscle biopsy was less likely to be performed when an affected sibling had already been biopsied, or if phenotype was suggestive of a diagnosis where muscle biopsy is often nonspecific such as in case of laminopathies. All subjects had undergone genetic testing (figure 1), with an average of five genes screened per family.

**Phenotype analysis**

The prevalence of observed clinical features in this undiagnosed group of patients is shown in Table 3. Onset of symptoms was mainly congenital (39.5%) with onset less frequently in childhood (29.4%) and adulthood (31%). Clinical features varied according to age of disease manifestation (figure 3). Of those patients with congenital onset disease, 63.8% had a normal serum CK level, while in adult-onset cases normal CK levels were present in only 13.5% of cases, with higher figures (37.8%) for values >1000IU/L. Weakness affecting proximal and distal muscles equally is the most common presentation in congenital onset disease (40.4%), while adult onset disease shows involvement of predominantly proximal compartments (64.9%). Distal weakness alone appears to be uncommon in all age groups. Normal strength was observed in 11 patients, referred to our service in view of the presence of features such as contractures, muscle hypertrophy, high serum CK or fatigue. Joint contractures and respiratory dysfunction are more prevalent in patients with younger age onset disease (70.2% and 55.3%, respectively) compared to adult onset (43.2% and 29.7%, respectively). The likely inheritance pattern observed varies with age of onset of disease, with patients with congenital onset disease mainly occurring sporadically, while 56.8% of adult onset cases showed a positive family history.

**Hierarchical cluster analysis**

Hierarchical cluster analysis of the 119 patients identified six clusters of patients in which a common phenotype is delineated (figure 1). Cluster 1 comprises 11 patients with no muscle weakness. Cluster 2 comprises 4 unrelated patients with likely dominantly inherited congenital onset disease, normal serum CK and a combination of contractures, scoliosis, respiratory and cardiac dysfunction. The third cluster comprises 26 patients, 2 of whom are first cousins, with congenital or childhood onset disease associated with a normal (or mildly elevated) serum CK level and an absence, in general, of contractures or rigid spine. Twenty nine patients, including two pairs of siblings, are delineated into a fourth cluster defined by the presence of joint contractures, often at more than 3 joint sites, and frequently accompanied by a combination of rigid spine, scoliosis and respiratory or cardiac dysfunction. Within cluster 4 the dendrogram indicates a further subdivision into a cohort (cluster 4-a) of predominantly congenital onset disease with normal serum CK, and cluster 4-b with variable age at onset and serum CK often >1000IU/L. A fifth cluster of 14 patients, including a sibling pair, is characterised by childhood or adult onset, normal or mildly elevated CK and a positive family history, consistent with dominant inheritance in 10 patients, and recessive inheritance in 4 patients. Twenty-two patients, including a sibling pair, comprise the sixth cluster, characterised by mainly proximal lower limb weakness manifesting in adulthood consistently with an elevated CK, often in excess of 1000IU/L, and recessive inheritance.

**Table 3: Numbers of patients exhibiting clinical features according to age of disease onset:** 

Clinical feature	Age of disease onset		
	congenital (<2 years)	Childhood (2-16 years)	Adult (>16years)
**Creatine Kinase level (IU/L)**			
Absent	8	8	6
Normal (<250)	30	13	5
250-999	8	8	12
1,000-2,999	0	4	13
3,000-9,999	1	1	1
>10,000	0	1	0
**Presence of any contractures (including rigid spine)**			
yes	33	18	16
no	14	17	21
**Presence of any respiratory dysfunction**			
yes	26	7	11
no	21	28	26
**Inheritance pattern**			
Recessive	4	7	6
Dominant	4	4	10
Sporadic	39	24	21
**Pattern of muscle involvement**			
Proximal	7	7	8
Proximal>distal	12	10	16
Proximal and distal	18	9	7
Distal>proximal	4	4	3
Distal	0	2	1
No weakness	6	3	2

**Dendrogram of the hierarchical cluster analysis of 119 undiagnosed patients with genetic muscle disease in the north of England:**
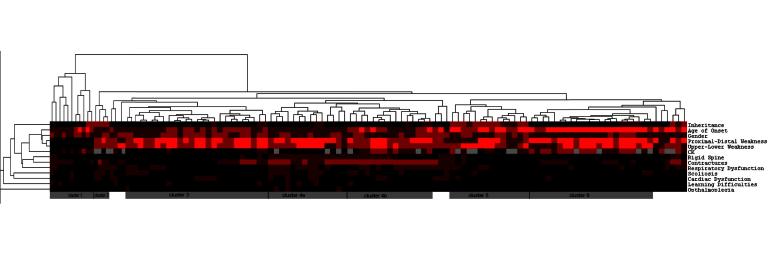
**Legend**: Each vertical column of coloured squares corresponds to an individual patient, and each row indicates a clinical feature. **Inheritance**: **black** indicates no other affected family members, **dark red** - recessive inheritance, **medium** **red** - dominant inheritance; **Age of Onset**: **dark red** – congenital, **medium red** – childhood, **bright red** – adult; **Gender**: **dark red** - male, **medium red** – female, **black** – both male and female affected; **Proximal/Distal Weakness**: **black** –no weakness, **dark red** – predominantly distal, **medium red** – proximal and distal equally affected, **bright red** – predominantly proximal; **Upper/Lower Weakness**: **black** –no weakness, **dark red** – predominantly upper, **medium red** – upper and lower equally affected, **bright red** – predominantly lower; **Contractures: black** – no joint contractures, **dark red** – contractures at 2 or less joint sites, **medium red** – contractures at 3 or more joint sites; **Serum CK**: **grey** – absent data, **black** – normal, **dark red** – 250-999IU/l, **medium red** – more than 1000IU/l; **Rigid Spine, Respiratory Dysfunction, Scoliosis, Cardiac Dysfunction, Learning Difficulties **and **** **Opthalmoplegia**: **black** – not present, **dark red** – present.

## Discussion

In this study we present an exploratory review of undiagnosed patients attending a regional genetic muscle clinic in the north of England. The intended benefits of this evaluation were to highlight areas of diagnostic difficulty, identify cases with a shared phenotype who may be amenable to collective diagnostic testing or research, and review the strengths and weaknesses of our current diagnostic strategy. In addition to manually reviewing the collated phenotype data, a hierarchical cluster analysis was performed to provide an unbiased interpretation of this data. This mathematical algorithm, first created by Sokal and Michener (1958), groups items into ‘clusters’ according to similarity, and has previously been used to identify constellations of clinical features which correspond with clinical variants of the neuromuscular disease SMARD 5 . We have used this as a tool to sort cases into groups according to the degree of similarity between individuals, on the basis that patients with a common phenotype may have the same underlying disease. This method has identified several interesting features of this population. In particular, the cluster analysis identified a large group of patients with a congenital onset, and normal or mildly elevated serum CK levels. This is in contrast to the finding by Norwood *et al* (2009)^3^ that congenital muscular dystrophies collectively accounted for only 18 patients, or 1.6% of the same clinic population. This finding confirms that these patients remain diagnostically challenging. We also identified a smaller group of patients (cluster 6, Fig. 1) with adult onset proximal weakness, significantly elevated CK (>1000IU/l) and possible recessive inheritance, suggesting the possible presence of a still unidentified autosomal recessive form of adult onset myopathy with high CK. Interestingly, adult onset disease with a serum CK that is normal or <1000IU/L, was found to be more likely to have a dominant family history (cluster 5, and other patients, in Fig. 1). In addition, distal phenotypes were found to be uncommon, in keeping with the clinical perception that the so-called distal myopathies are rare. Cluster analysis also identified a group with prominent contractures and skin changes, some of whom also had collagen VI abnormalities on skin fibroblast immunofluorescence analysis^6^ , consistent with a collagenopathy diagnosis, who are negative for collagen VI mutations, supporting the likelihood of a significant degree of genetic heterogeneity in collagen VI disorders^7^.

Our current diagnostic strategy involves gene-by-gene testing on the basis of a patient’s presenting phenotype. However this method is limited by the clinical and genetic heterogeneity of genetic muscle diseases and the time required for each test to be sequentially performed can result in a long and frustrating wait for diagnosis. This approach may be improved by more accurate phenotyping, for example incorporating the use of muscle magnetic resonance imaging to delineate the pattern of muscle involvement and thus inform selective gene testing^8^ . However, our study shows that despite in depth clinical analysis and accurate genetic investigations, with analysis of up to 10 genes per families, a proportion of muscle patients with clearly defined phenotypes remain undiagnosed.

The increasing availability of NGS will dramatically change the diagnostic approaches to patients in whom a genetic diagnosis is still missing. Indeed these methods have already identified novel genetic causes of muscular dystrophies, such as TTN^9^ and DNAJB6^10^ and have also been applied to achieve a diagnosis in families with autosomal dominant limb-girdle muscular dystrophy^11^ .We believe that this analysis and the phenotypic characterisation it brings to this group of patients is a useful preparatory step for the application of NGS techniques, either by using the database created in this study to identify patients for testing of novel genes identified by NGS, or for selection of clusters of phenotypically similar patients for exomic or genomic sequencing. In singleton cases NGS would allow testing of multiple genes based on recognition of a more broad phenotypic category (such as all known LGMD genes for patients with proximal weakness), significantly curtailing the diagnostic odyssey that these patients currently undergo. In addition this will readily exclude known genes to aid identification of patients in whom further research efforts may help in identifying novel disease genes. We anticipate that the correlation of detailed phenotypic data with NGS will be required to extend the benefits of a molecular diagnosis to all patients with genetic muscle disease.

## Competing Interests

The authors declare no competing interest exists.
